# Corneal Confocal Microscopy to Image Small Nerve Fiber Degeneration: Ophthalmology Meets Neurology

**DOI:** 10.3389/fpain.2021.725363

**Published:** 2021-08-19

**Authors:** Ioannis N. Petropoulos, Gulfidan Bitirgen, Maryam Ferdousi, Alise Kalteniece, Shazli Azmi, Luca D'Onofrio, Sze Hway Lim, Georgios Ponirakis, Adnan Khan, Hoda Gad, Ibrahim Mohammed, Yacob E. Mohammadi, Ayesha Malik, David Gosal, Christopher Kobylecki, Monty Silverdale, Handrean Soran, Uazman Alam, Rayaz A. Malik

**Affiliations:** ^1^Department of Medicine, Weill Cornell Medicine-Qatar, Doha, Qatar; ^2^Department of Ophthalmology, Meram Faculty of Medicine, Necmettin Erbakan University, Konya, Turkey; ^3^Faculty of Biology, Medicine and Health, University of Manchester, Cardiovascular Trials Unit, Manchester University NHS Foundation Trust, Manchester, United Kingdom; ^4^Centre for Diabetes, Endocrinology and Metabolism, Manchester University NHS Foundation Trust, Manchester, United Kingdom; ^5^Department of Experimental Medicine, Sapienza University, Rome, Italy; ^6^Department of Neurology, Salford Royal National Health System (NHS) Foundation Trust, Manchester Academic Health Sciences Centre, University of Manchester, Manchester, United Kingdom; ^7^Department of Cardiovascular and Metabolic Medicine, Clinical Sciences Centre, Pain Research Institute, Institute of Life Course and Medical Sciences, University of Liverpool, Liverpool University Hospital National Health System (NHS) Foundation Trust, Liverpool, United Kingdom

**Keywords:** corneal confocal microscopy, neurodegeneration, painful neuropathy, diabetes, biomarker

## Abstract

Neuropathic pain has multiple etiologies, but a major feature is small fiber dysfunction or damage. Corneal confocal microscopy (CCM) is a rapid non-invasive ophthalmic imaging technique that can image small nerve fibers in the cornea and has been utilized to show small nerve fiber loss in patients with diabetic and other neuropathies. CCM has comparable diagnostic utility to intraepidermal nerve fiber density for diabetic neuropathy, fibromyalgia and amyloid neuropathy and predicts the development of diabetic neuropathy. Moreover, in clinical intervention trials of patients with diabetic and sarcoid neuropathy, corneal nerve regeneration occurs early and precedes an improvement in symptoms and neurophysiology. Corneal nerve fiber loss also occurs and is associated with disease progression in multiple sclerosis, Parkinson's disease and dementia. We conclude that corneal confocal microscopy has good diagnostic and prognostic capability and fulfills the FDA criteria as a surrogate end point for clinical trials in peripheral and central neurodegenerative diseases.

## CCM Technique

### Confocal Microscopy: A Brief History

Minsky invented the first “double focusing stage scanning microscope” in 1955, which Petran evolved into a functional confocal microscope in 1960. The first clinical corneal confocal microscope was built by Dilly in 1988 and was used to image the corneal epithelium, endothelium and stromal keratocytes of conscious humans. The corneal confocal microscope (CCM) is currently used by ophthalmologists to assess epithelial and stromal abnormalities and acanthamoeba infection (1). We will highlight how this ophthalmic tool has been increasingly utilized to quantify C-fibers in peripheral and central neurodegenerative diseases.

### Corneal Confocal Microscopes

**Figure 1:**
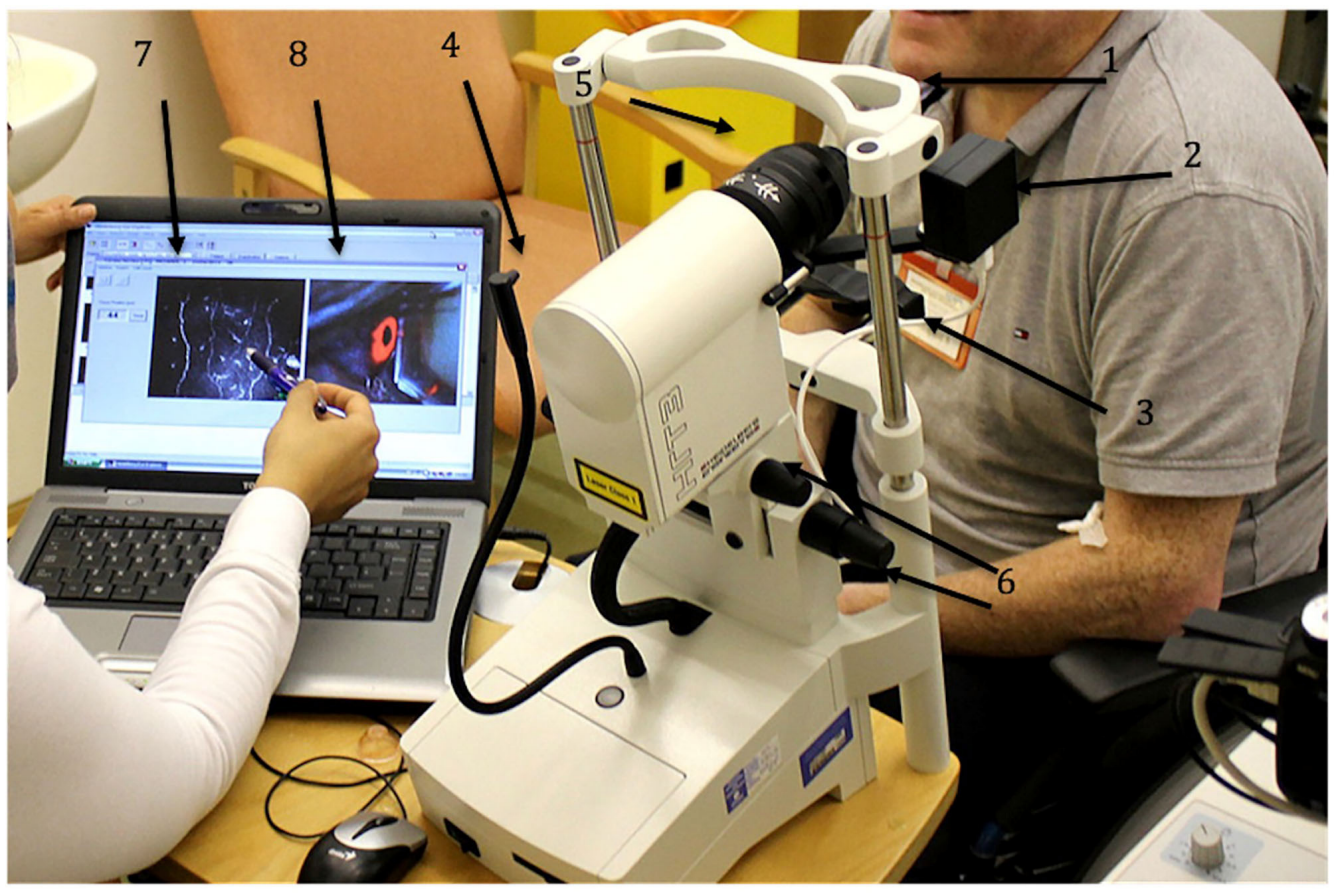
HRT III-RCM Corneal confocal microscope (CCM). 1 – forehead bar, 2 – CCD camera, 3 – chin rest, 4 – fixation target, 5 – corneal module, 6 – knobs to align the CCM, 7 – CCM live image, 8 – CCD camera live image.

The laser scanning CCM HRTIII (Heidelberg Retina Tomograph III Rostock Corneal Module, Heidelberg Engineering GmbH, Heidelberg, Germany) is the most commonly used instrument which utilizes a single wavelength (670 nm red) Helium-Neon Diode class 1 laser to generate high resolution images of the corneal epithelial cells, keratocytes, endothelial cells, sub-basal nerve plexus (Figure 1) and dendritic cells which are antigen-presenting cells that have been found to be increased in a number of autoimmune and inflammatory neuropathies. Other commercially available slit scanning *in vivo* CCM's are manufactured by Tomey Corporation (Cambridge, MA, USA), Nidek Technologies (Gamagori, Japan) and Helmut Hund (Wetzlar, Germany), but have limited image resolution for the sub-basal nerve plexus.

### CCM Image Acquisition

Images can be captured using the section, volume or sequence modes. The section mode allows the examiner to manually focus the field of view on the area of interest and is more suitable for experienced users. The sequence and volume modes enable faster automated sequential image acquisition but have limited image quality. Most studies have analyzed 5–8 high-quality, non-overlapping images from the central cornea (2). A perceived limitation of CCM is the small field of view, therefore some centers have used wide field imaging to create maps of the subbasal nerve plexus (3).

### CCM Image Quantification

The main corneal nerve morphological parameters quantified include corneal nerve fiber density (CNFD), branch density (CNBD), fiber length (CNFL) and inferior whorl length (IWL) (4). CNFD refers to the total number of main nerve fibers in a CCM image (fibers/mm^2^), CNBD is defined by the number of primary branches arising from the main nerve fibers (branches/mm^2^), CNFL is the total length of all nerve fibers and branches in a CCM image (mm/mm^2^) and IWL is the total length of nerves at the inferior whorl. Corneal nerve fractal dimension is a mathematical derivation of the pattern of corneal nerves and may allow the differentiation of neuropathies of different etiology (5). CCMetrics and ACCMetrics are freely available software for manual and automated quantification of sub-basal corneal nerves, respectively (6). CCMetrics also has been used to manually count the density of corneal dendritic cells (no./mm^2^). Novel Artificial Intelligence (AI) based algorithms have also been developed for fully automated corneal nerve quantification (7) and identification of patients with and without diabetic neuropathy (8).

## CCM in Peripheral Neuropathies

### Diabetic Peripheral Neuropathy

**Figure 2:**
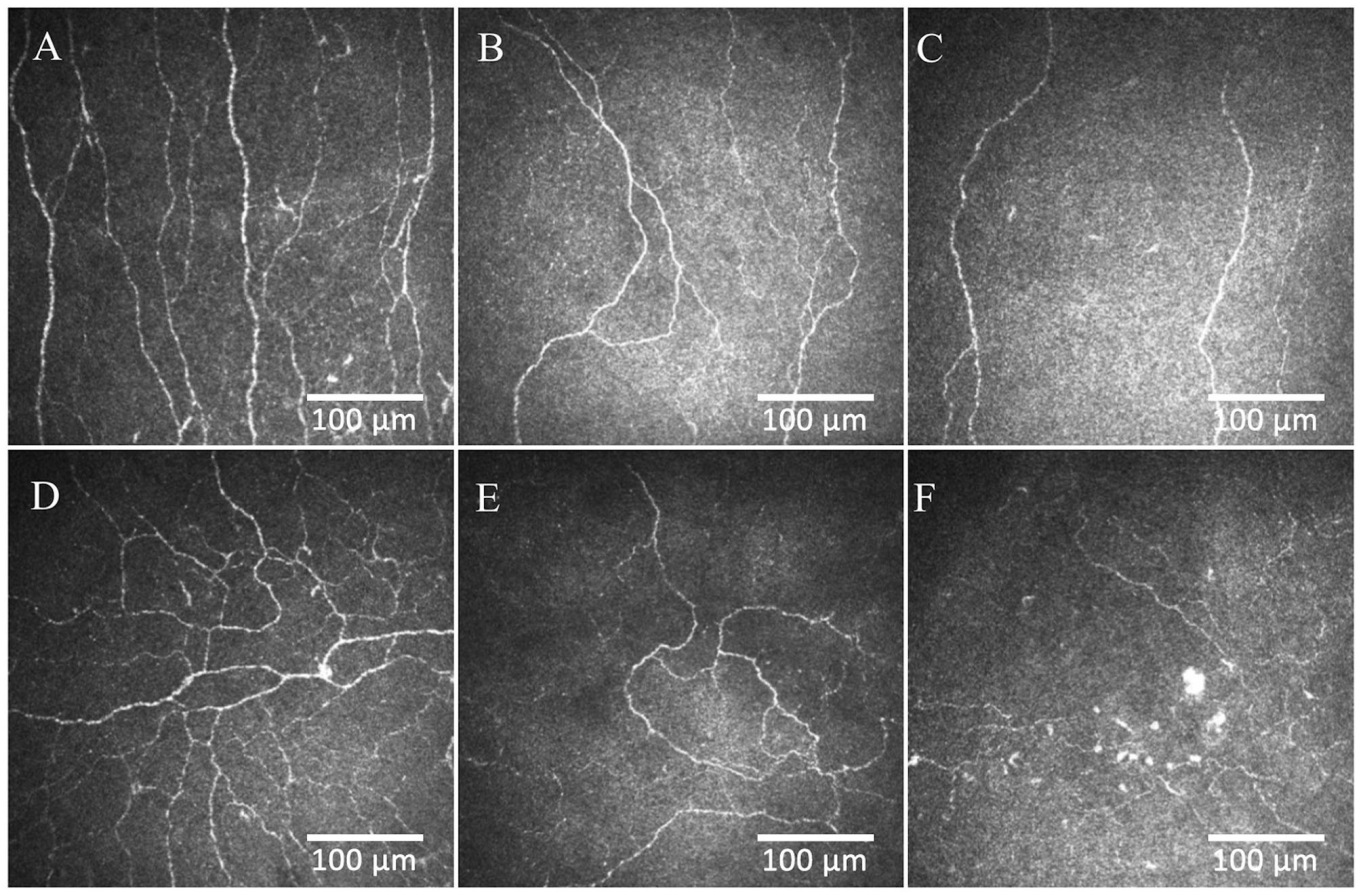
CCM images of the central cornea and inferior whorl in a healthy control **(A,D)**, patient with painless **(B,E)** and painful **(C,F)** diabetic neuropathy.

We pioneered the use of CCM as a measure of neuropathy in 2003, by showing early and progressive loss of corneal nerve fibers in patients with increasing severity of diabetic neuropathy (9). Quattrini et al. (10) subsequently showed a comparable reduction in corneal nerve and intraepidermal nerve fiber density in DPN. Chen et al. (11) showed that CNFD had a superior diagnostic performance for DPN compared to intra epidermal nerve fiber density (IENFD) and this was confirmed in our subsequent study (12). The NIH consortium study (13) of 998 subjects with type 1 and type 2 diabetes reported a 0.88/0.88 sensitivity/specificity for corneal nerve fiber length in the diagnosis of DPN. Age-adjusted normative values for CCM show a small but progressive loss of corneal nerves with increasing age (14). More recently we have shown that the severity of and risk factors associated with corneal nerve loss differ between patients with type 1 and type 2 diabetes with an association between LDL cholesterol and triglycerides in type 1 and age, HbA1c and weight in type 2 diabetes (15). Corneal nerve loss also occurs in the early stages of diabetes in children (16, 17) and adults (18) with type 1 diabetes before the development of diabetic retinopathy and microalbuminuria and in subjects with impaired glucose tolerance (19, 20) and recently diagnosed type 2 diabetes (21). Patients with painful diabetic neuropathy show greater corneal nerve loss particularly at the inferior whorl (22, 23) (Figure 2). Studies from Italy (24), China (25), Japan (26), New Zealand (27) and the UK (28) show corneal nerve loss in patients with diabetic autonomic neuropathy and we have shown that corneal nerve loss occurs in patients with diabetes and obesity with erectile dysfunction (29, 30). Reduced corneal nerve fiber length predicts 4-year incident DPN (31, 32) and a more rapid decline in CNFL has been associated with the development of DPN (33) and foot ulceration (34).

### Chemotherapy-Induced Peripheral Neuropathy

Chemotherapy-induced peripheral neuropathy (CIPN) is associated with pain, which may lead to dose reduction or discontinuation of chemotherapy. An early case-report showed that a patient with colorectal cancer treated with capecitabine had a reduction in corneal nerves with increased sprouting, indicative of concomitant nerve degeneration and regeneration (35). We have shown corneal nerve loss in a cohort of patients with gastro-esophageal cancer followed by an increase in corneal nerve fiber length after the 3rd cycle of platinum-based chemotherapy, indicative of nerve regeneration (36). Recently in patients with breast and colon cancer, 5 years after the initial development of CIPN, there was no change in corneal nerve morphology (37). However, a reduction in central and inferior whorl corneal nerve fiber length has been found in patients 3–24 months after treatment with paclitaxel or oxaliplatin (38). The association between changes in corneal nerve morphology and manifestations of CIPN are complex, appear to be temporally related and demand further study.

### Inflammatory Neuropathies

Behcet's disease is a chronic relapsing vascular inflammatory disease with a number of neurological manifestations, including peripheral neuropathy. We have recently shown corneal nerve loss and a significant increase in dendritic cell density (39). Schneider et al. (40) showed a significant reduction in subbasal nerve fiber density and length with an increase in dendritic cell density in patients with chronic inflammatory demyelinating polyneuropathy (CIDP). Stettner et al. (41) confirmed and extended these results by showing significant corneal nerve loss and an increase in dendritic cells in a large cohort of patients with CIDP, multifocal motor neuropathy (MMN) and monoclonal gammopathy of unknown significance (MGUS) which was associated with neurologic severity and the presence of pain. Moreover, whilst the extent of corneal nerve fiber loss was comparable, dendritic cell density in proximity to nerve fibers was found to be increased in patients with CIDP compared to diabetic neuropathy (42).

### Human Immunodeficiency Virus Neuropathy

Neuropathic pain is a frequent and debilitating manifestation of Human Immunodeficiency Virus (HIV) infection. Kemp et al. (43) showed a reduction in corneal nerve fibers in patients with HIV-associated neuropathy and we also showed that corneal nerve fractal dimension differs between patients with HIV neuropathy and other peripheral neuropathies (44).

### Idiopathic Small Fiber Neuropathy (ISFN)

Idiopathic small fiber neuropathy is characterized by painful neuropathic symptoms and small fiber dysfunction/damage with preserved large nerve fiber function (45). In a cohort of patients with ISFN we previously showed a significant reduction in corneal nerve density and length and related it to sensory symptoms (46). In a case-control study, 86 patients with SFN underwent neurological examination, quantitative sensory testing, distal and proximal skin punch biopsy, and sub-group of 55 patients additionally underwent pain-related evoked potentials (PREP), corneal confocal microscopy (CCM), and a quantitative sudomotor axon reflex test (QSART). An abnormal distal intraepidermal nerve fiber density (IENFD) (60/86, 70%) and neurological examination (53/86, 62%) identified those with SFN and CCM and/or PREP further increased the proportion of patients identified with SFN to 85%, whilst QST, QSART, and proximal IENFD contributed minimally (47).

### Hereditary Neuropathy

Corneal nerve loss has been reported in patients with Charcot Marie Tooth Disease Type 1A (48) and a hereditary neuropathy with a rare nerve growth factor-β mutation (49) where the severity of corneal nerve loss was related to the reported pain intensity. We have also demonstrated corneal nerve loss in patients with Friedreich's ataxia, a multi-system autosomal recessive disease caused by homozygous guanine-adenine-adenine (GAA) repeat expansions within intron 1 of the frataxin gene. The severity of corneal nerve loss has been related to the number of GAA repeats and clinical disability assessed using the Scale for the Assessment and Rating of Ataxia and Friedreich's Ataxia Rating Scale (50). In a cohort of 51 patients with neurofibromatosis type 1, 8% had abnormal nerve conduction studies, 13% had abnormal thermal thresholds, 22% had abnormal intraepidermal nerve fiber density, however, 52% had reduced corneal nerve fiber length (51).

### Amyloid Neuropathy

Transthyretin familial amyloid polyneuropathy (TTR-FAP) is a fatal inherited disorder characterized by pain, numbness and weakness due to a progressive neuropathy (52). Small fiber neuropathy with loss of intra-epidermal nerve fibers (53) and altered thermal thresholds (54) are key features of TTR-FAP. CCM showed corneal nerve fiber loss in a patient with light chain amyloid neuropathy secondary to multiple myeloma (55). In a series of 15 patients with TTR-FAP a reduction in corneal nerve fiber length was related to the neuropathy impairment score of the lower limbs, autonomic dysfunction, sensory nerve action potential and IENFD (56). CNFL could be measured in all participants, whilst sural nerve amplitude and IENFD could only be measured in 73 and 27% of patients, respectively. This lack of a floor effect increases the utility of CNFL compared to IENFD in longitudinal and interventional studies of amyloid neuropathy. Recently, a study from China has confirmed and extended these findings by showing corneal nerve loss in the central and inferior whorl regions with an AUC for CNFL and IWL of 88.0 and 89.3%, respectively, for the diagnosis of familial amyloid neuropathy (57).

### Fabry Disease

Painful neuropathy is a hallmark of Fabry's disease due to the accumulation of globotriaosylceramide (GI_3_) leading to nerve damage (58, 59). We were the first to report corneal nerve loss using a first generation Tomey ConfoScan in patients with Fabry disease (60). More recently using a HRT III CCM device we have extended these observations and confirmed corneal nerve loss and an increase in dendritic cells which correlated with the total Mainz severity score index (61).

### Hypothyroidism

In patients with primary hypothyroidism and in patients with hyperthyroidism undergoing radioiodine therapy, corneal nerve fiber density was reduced and improved after 12 months of treatment with levothyroxine (62).

### Fibromyalgia

Pain is a major feature of fibromyalgia and several studies have shown corneal nerve fiber loss in patients with fibromyalgia. In an early study from Mexico, stromal nerve thinning and a reduction in subbasal nerve fiber density was related to a variety of pain descriptors (63). In a subsequent very detailed phenotyping study from the Netherlands, corneal nerve loss was identified in 51% of patients with fibromyalgia and related to central sensitization (64). A study from Turkey reported a reduction in corneal nerve fiber length which correlated with the “widespread pain index” in patients with fibromyalgia (65). More recently in a large cohort of 117 women with fibromyalgia we have demonstrated multiple small fiber abnormalities including a comparable reduction in IENFD and corneal nerves (66).

### Post-COVID Neuropathy

**Figure 3:**
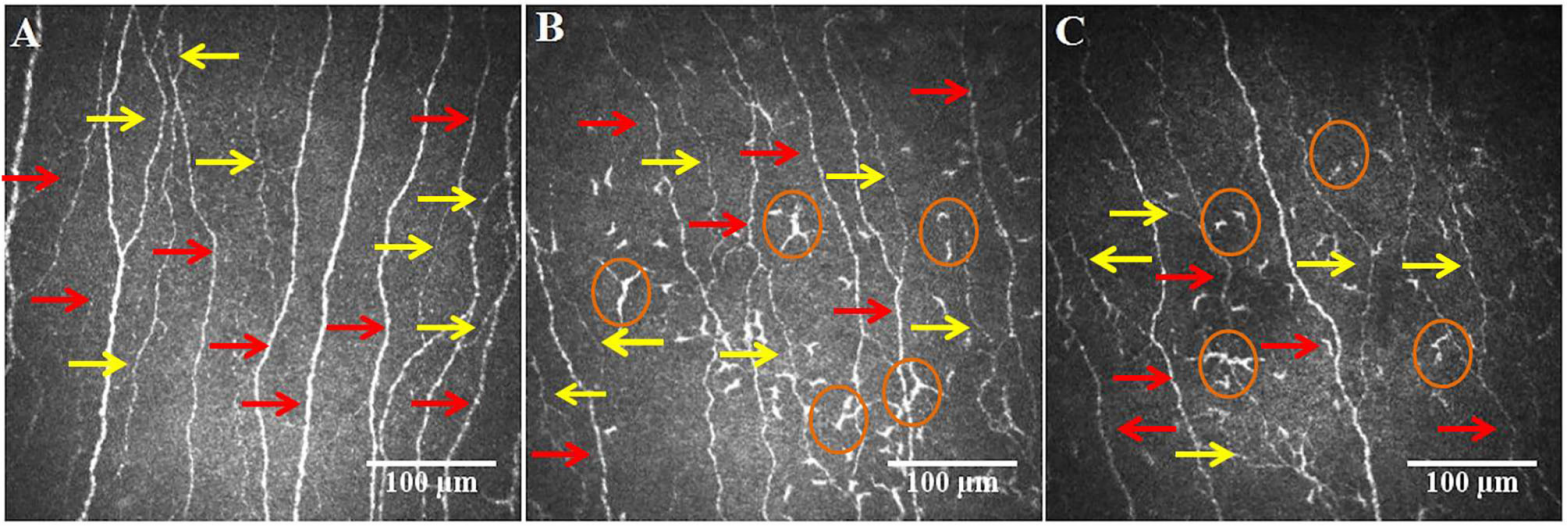
CCM images of the central cornea in a healthy control **(A)**, post-COVID-19 patient without **(B)** and with **(C)** long-COVID, showing a loss of main nerves (red arrows) and branches (yellow arrows) in the patient with long-COVID and an increase in dendritic cells (circles) in patients with and without long-COVID.

In a small series of 4 patients who developed painful diabetic neuropathy during acute SARS-CoV 2 infection we recently showed evidence of altered taste and smell and increased thermal thresholds, suggestive of underlying small fiber neuropathy (67). There are small studies which indicate both large (68) and small (69) fiber neuropathy in patients following COVID-19, although this may reflect nerve damage associated with severe disease and critical illness (70, 71). However, we have recently shown corneal nerve loss and increased dendritic cells in a cohort of patients 12 weeks after relatively mild COVID-19, particularly those with neuropathic and fibromyalgia like symptoms who fulfilled the criteria for long-COVID (Figure 3) (72).

## CCM in Central Neurodegenerative Diseases

An increasing number of centers have explored the utility of corneal nerve loss as a surrogate marker of neurodegeneration in central neurodegenerative diseases.

### Parkinson's Disease

**Figure 4:**
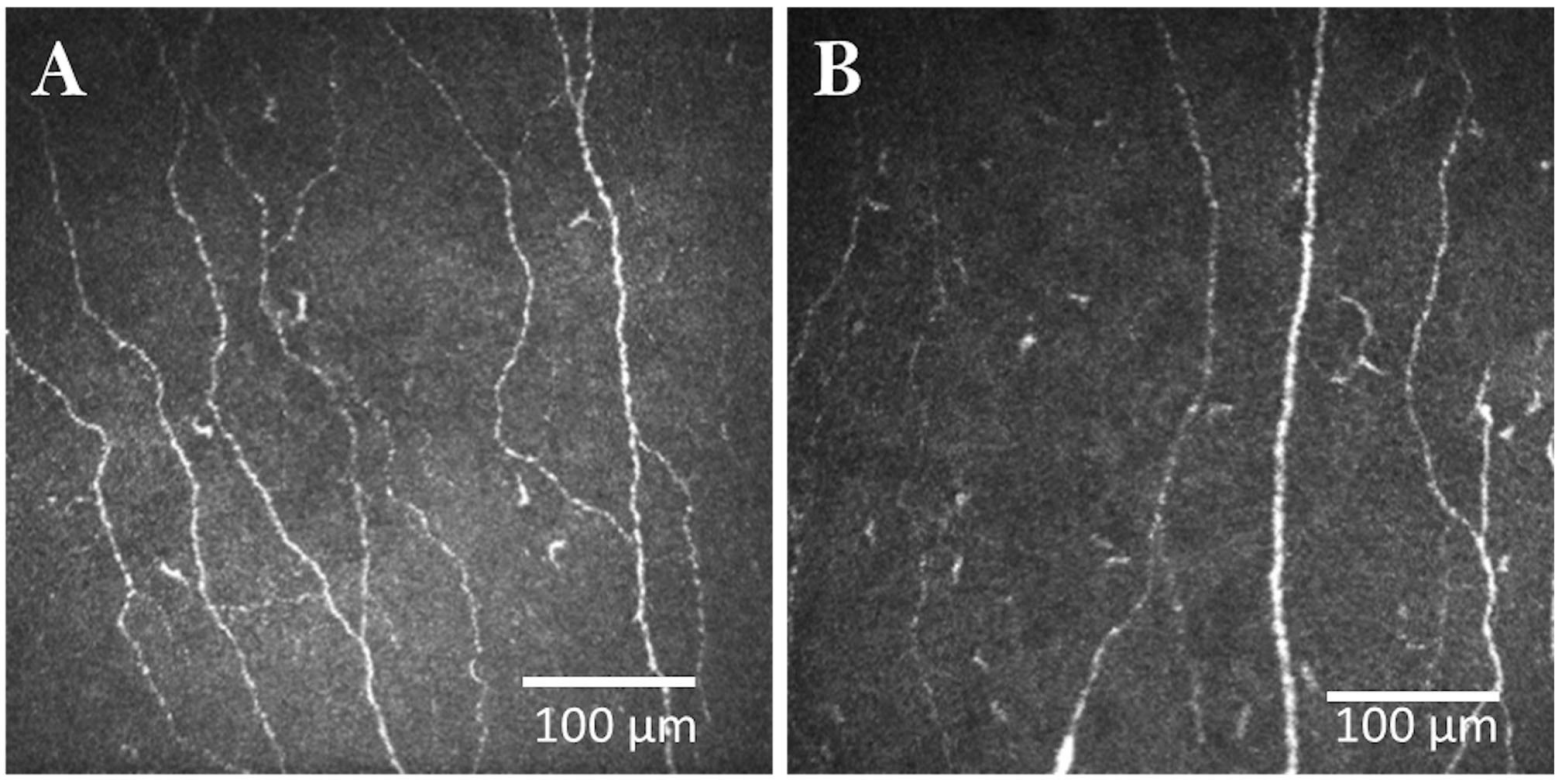
CCM images of the central cornea in a healthy control **(A)** and patient with Parkinson's disease **(B)**.

Pain is an increasingly recognized non-motor feature of PD (73). A study of 25 patients with PD showed reduced corneal sensitivity and a reduction in corneal nerve fiber density, branch density and length which was related to therapy with dopaminergic therapy (74). Kass-Iliyya et al. (75) showed corneal nerve loss in patients with PD which was related to the unified PD rating scale and autonomic dysfunction. Another study in 26 newly diagnosed patients with PD showed a reduction in corneal nerve parameters, with normal nerve conduction and IENFD (76). Corneal nerve loss has also been related to severity of cognitive dysfunction in patients with Parkinson's disease (77) and associated with altered white matter diffusion properties of the trigeminal nerve (78). Recently, a significant decrease in the directional anisotropy coefficient and an increase in the directional symmetry coefficient of corneal nerve fibers has been demonstrated in patients with PD (79). We have confirmed the loss of corneal nerve fibers in a large cohort of 98 participants with PD (80) (Figure 4). In a recent study from China, CNFD showed an excellent diagnostic performance with a AUC of 0.96 for PD and corneal nerve fiber parameters correlated with the severity of motor symptoms measured using the H-Y stage, UPDRS-III and UPDRS-Total (81). Furthermore, we have shown that a lower corneal nerve fiber length predicts progressive worsening of UPDRS-III over 12 months in patients with PD (82). CCM could therefore add to the diagnostic toolbox for pre-motor Parkinson's disease.

### Multiple Sclerosis

Common initial symptoms of MS include hypoesthesia, dysesthesia, paraesthesia and mononuclear painful visual loss (83, 84) and of course Lhermitte's sign is characterized by a transient electric shock sensation extending down the spine and/or extremities upon flexion of the neck (85). The etiology of pain is heterogeneous but includes central neuropathic pain, which can occur in almost a third of patients with MS and has been associated with increased thermal thresholds (86). Trigeminal neuralgia is a troubling feature of multiple sclerosis, and a recent diffusion tensor imaging study has shown that patients with a poor response to treatment have lower fractional anisotropy and higher radial diffusivity of the pontine trigeminal fibers (87). Several studies (88–90) have demonstrated a significant reduction in subbasal corneal nerve density and a recent study has shown altered pupillary responses (91) in patients with multiple sclerosis. Corneal nerve loss correlates with disease severity and an increase in corneal immune cells (89, 92). More recently a longitudinal study over 2 years has shown that progressive corneal nerve loss is associated with worsening neurological disability (93). However, no study to date has assessed the relationship between pain and corneal nerve loss.

### Amyotrophic Lateral Sclerosis

Pain is a neglected feature of ALS (94) but can arise in relation to cramps, spasticity and neuropathy and may even occur before the first motor symptom (95). Corneal nerve loss has been demonstrated in a small cohort of patients with ALS and associated with the bulbar function disability score (96).

### Stroke

Central post-stroke pain has been estimated to occur in 8–55% of stroke patients and may be constant or intermittent neuropathic pain accompanied by dysesthesia of temperature and/or pressure sensations within the area of the body corresponding to the stroke lesion (97). The etiology of this condition is poorly understood and it is often resistant to conventional pharmacological treatment options (98). Corneal nerve loss has been demonstrated in patients with Transient Ischemic Attack (TIA), minor stroke (99) and major stroke (100), with greater severity in patients with recurrent stroke (101) and it is associated with the presence of white matter hyperintensities, markers of small vessel disease (102). The relationship between corneal nerve loss and central post-stroke pain has not been explored.

### Dementia

Pain is highly prevalent in patients with dementia (103), however it is not routinely assessed and is poorly managed (104). There is a growing interest for the role of CCM as a biomarker of neurodegeneration in subjects with dementia (105). We have shown a significant reduction in corneal nerve fibers in patients with mild cognitive impairment and dementia (106, 107). We have also recently shown stromal nerve loss with preservation of sub-basal nerves in patients with front-temporal dementia (108).

### Migraine

Migraine is characterized by severe headache accompanied by nausea and sensitivity to light. In patients with migraine corneal nerve fiber density and length were reduced (109) and Shetty et al. demonstrated corneal nerve loss in patients with chronic migraine and photophobia, but not in patients without photophobia (110).

### Trigeminal Neuralgia

Trigeminal neuralgia manifests with severe often excruciating electric shock-like pain affecting the lower jaw and face. Interestingly, corneal nerve fiber density and length were reduced in both the ipsilateral and contralateral cornea of patients with trigeminal neuralgia and there was no difference between patients with and without nerve vessel conflict (111).

### Burning Mouth Syndrome

Burning mouth syndrome is a relatively rare condition which occurs more frequently in older women and is characterized by a burning feeling on the roof of the mouth, tongue and lips. In a study of 17 patients with BMS there was a significant reduction in corneal nerve fiber density and length and an increase in dendritic cell density (112).

### CCM in Clinical Trials

Mehra et al. (113) showed that CCM can detect early nerve fiber repair with an increase in corneal nerve fiber density and length following normalization of glycaemia and renal function after simultaneous pancreas and kidney (SPK) transplantation. Tavakoli et al. (114) showed an improvement in CCM parameters 12 months after SPK transplantation with no change in symptoms and deficits, neurophysiology, quantitative sensory testing and skin biopsy. More recently we have shown an early (12 months) and continued improvement in corneal nerve fiber length which was associated with an improvement in neuropathic symptoms and neurophysiology at 36 months (115). We have also recently shown that bariatric surgery leads to corneal nerve regeneration (116). A novel first-in-class peptide (ARA290-Cibinetide) which reduces inflammation was associated with an increase in corneal nerve fiber density and length in patients with sarcoidosis-related neuropathy (117, 118) and T2DM (119) and was paralleled by an improvement in pain scores and functional outcomes. In a subsequent Phase 2b study, the improvement in corneal nerve morphology correlated with the expression of GAP-43^+^ intraepidermal nerve fibers, indicating nerve fiber repair and an improvement in pain intensity after 28 days (118). In a trial of seal oil omega-3 polyunsaturated fatty acid in patients with T1DM there was a significant 29% increase in CNFL, with no change in nerve conduction velocity and sensory function over 12 months (120) which was associated with higher baseline omega-3 levels (121). More recently, a randomized placebo controlled trial has confirmed that treatment with long chain omega-3 supplements over 6 months was associated with an increase in corneal nerve fiber length indicative of corneal nerve regeneration, without improvement in neurophysiology or thermal thresholds in patients with type 1 diabetes (122). Recently we have shown an improvement in corneal nerve parameters in a randomized clinical trial of once weekly GLP-1 or insulin in patients with poor glycemic control (123). Greater nerve regeneration was observed, especially in patients without insulin resistance (124) and those with painful diabetic neuropathy (125).

## Conclusion

Regulatory agencies and pharmaceutical companies need to consider the compelling case for CCM as a marker of neurodegeneration and regeneration and as an end-point in clinical trials of new therapies in peripheral and central neurodegenerative diseases (1, 126).

## Author Contributions

HS has contributed significantly to the body of knowledge generated in this review by performing literature searches and reviewing the first draft. All authors contributed to the literature search and first draft of the review.

## Conflict of Interest

The authors declare that the research was conducted in the absence of any commercial or financial relationships that could be construed as a potential conflict of interest. There is no conflict of interest related to this work for any of the authors.

## Publisher's Note

All claims expressed in this article are solely those of the authors and do not necessarily represent those of their affiliated organizations, or those of the publisher, the editors and the reviewers. Any product that may be evaluated in this article, or claim that may be made by its manufacturer, is not guaranteed or endorsed by the publisher.
